# Methylation and Expression of Immune and Inflammatory Genes in the Offspring of Bariatric Bypass Surgery Patients

**DOI:** 10.1155/2013/492170

**Published:** 2013-06-11

**Authors:** Frédéric Guénard, André Tchernof, Yves Deshaies, Katherine Cianflone, John G. Kral, Picard Marceau, Marie-Claude Vohl

**Affiliations:** ^1^Department of Food Science and Nutrition, Institute of Nutrition and Functional Foods (INAF), Laval University, Québec, QC, Canada G1V 0A6; ^2^Endocrinology and Nephrology, CHU de Québec Research Center, Québec, QC, Canada G1V 4G2; ^3^Québec Heart and Lung Institute, Québec, QC, Canada G1V 4G5; ^4^Department of Medicine, Laval University, Québec, QC, Canada G1V 0A6; ^5^Department of Surgery, SUNY Downstate Medical Center, Brooklyn, NY 11203, USA; ^6^Department of Surgery, Laval University, Québec, QC, Canada G1V 0A6

## Abstract

*Background*. Maternal obesity, excess weight gain and overnutrition during pregnancy increase risks of obesity, type 2 diabetes mellitus, and cardiovascular disease in the offspring. Maternal biliopancreatic diversion is an effective treatment for severe obesity and is beneficial for offspring born after maternal surgery (AMS). These offspring exhibit lower severe obesity prevalence and improved cardiometabolic risk factors including inflammatory marker compared to siblings born before maternal surgery (BMS). *Objective*. To assess relationships between maternal bariatric surgery and the methylation/expression of genes involved in the immune and inflammatory pathways. *Methods*. A differential gene methylation analysis was conducted in a sibling cohort of 25 BMS and 25 AMS offspring from 20 mothers. Following differential gene expression analysis (23 BMS and 23 AMS), pathway analysis was conducted. Correlations between gene methylation/expression and circulating inflammatory markers were computed. *Results*. Five immune and inflammatory pathways with significant overrepresentation of both differential gene methylation and expression were identified. In the IL-8 pathway, gene methylation correlated with both gene expression and plasma C-reactive protein levels. *Conclusion*. These results suggest that improvements in cardiometabolic risk markers in AMS compared to BMS offspring may be mediated through differential methylation of genes involved in immune and inflammatory pathways.

## 1. Introduction

Parental obesity increases the risk of obesity in offspring through genetic, biological, and environmental influences [1–3]. Maternal obesity, weight gain, and increased body mass index (BMI) between pregnancies and gestational diabetes increase risks of offspring obesity, type 2 diabetes mellitus (T2DM), and cardiovascular disease (CVD) in the offspring [4–7]. Consistent with the chronic low-grade inflammatory state of obesity, elevated concentrations of interleukin-6 (IL-6) and C-reactive protein (CRP) have been observed in obese pregnant women [8]. Higher maternal levels of IL-6 were predictive of the increased growth and adiposity in the offspring [9]. Maternal diet-induced obesity in sheep resulted in higher fetal triglyceride (TG) levels [10] and upregulated inflammatory signaling [11]. Offspring of rats injected with IL-6 throughout pregnancy had an altered inflammatory profile associated with greater body fat mass and reduced insulin sensitivity [12].

Epigenetic processes are important mediators of early-life environment, metabolism, and body composition in offspring. Epigenetic changes in genes involved in the inflammatory response have been observed in response to maternal diet and adiposity. Differences in methylation levels of the peroxisome proliferator-activated receptor alpha (PPARA) and glucocorticoid receptor promoters have been observed in the liver of offspring of rats fed with protein-restricted diet during pregnancy [13, 14]. Methylation status of retinoid X receptor alpha (RXRA) promoter in umbilical cord tissue DNA was correlated with increased adiposity in children of mothers with lower carbohydrate intake [15]. Epigenetic mechanisms have been involved in inflammation and associated disorders including obesity [15, 16].

Bariatric surgery is known to improve glucose and lipid metabolism [17–19] and prevent arterial hypertension and type 2 diabetes [19–21]. Bariatric surgery also decreases the low-grade inflammation associated with obesity [22]. In previous studies, we demonstrated that children born after maternal bariatric surgery (AMS) exhibited lower prevalence of severe obesity, greater insulin sensitivity, and improved lipid profile in comparison to siblings born before maternal surgery (BMS) [23, 24]. In addition, AMS children demonstrated differences in a marker of chronic low-grade inflammation, namely the plasma C-reactive protein (CRP) [24].

This study was designed to analyze the relationship between maternal biliopancreatic diversion surgery and methylation levels of genes involved in immunologic and inflammatory pathways in BMS and AMS offspring. Pathway analyses were conducted using both methylation and expression data. Correlation analyses between gene methylation, expression, and plasma CRP levels were conducted for the IL-8 pathway.

## 2. Material and Methods

### 2.1. Subjects

Women from Québec City and surrounding areas (administrative regions of Capitale-Nationale, Mauricie, and Chaudière-Appalaches) who had given birth before and after biliopancreatic diversion with duodenal switch for severe obesity were eligible for the current study. A subset of 20 unrelated mothers was recruited along with 50 siblings born before and after surgery (25 BMS and 25 AMS offspring). Mothers and offspring attended the Québec Heart and Lung Institute (Québec City, Québec, Canada) or a regional hospital for assessment between July and October 2010. There were 17 mothers with siblings born before and after surgery (23 BMS and 24 AMS), two with BMS offspring only (2 BMS), and one mother with only one AMS offspring. This study was approved by the Québec Heart and Lung Institute Ethics Committee. Written informed consent was obtained from adults and mothers with assents from minors.

Maternal presurgical data were obtained from medical records. Percent body fat was determined for individuals at 6 years or more (BMS, *N* = 23; AMS, *N* = 17) using bioelectric impedance analysis (Tanita, Arlington Heights, IL, USA). Weight, height, and resting systolic (SBP) and diastolic (DBP) blood pressure were obtained using standardized procedures and measured at the interview. BMI was calculated for mothers, and BMI percentile for children was obtained from the National Health and Nutrition Examination Survey 2000 charts [25]. BMI *Z*-score was calculated for children using charts from the Centers for Disease Control and Prevention [26]. Severe obesity in offspring was defined using age- and sex-corrected adiposity measurements (BMI *Z*-score > 3; BMI percentile > 98%). Blood samples were collected from mothers and offspring after an overnight fast into BD Vacutainer tubes containing EDTA and PAXgene Blood RNA collection tubes (Qiagen, Valencia, CA, USA). Plasma TG, high-density lipoprotein cholesterol (HDL-C), low-density lipoprotein cholesterol (LDL-C), total cholesterol (total-C), total-C/HDL-C ratio, glucose, and insulin concentrations were measured as previously described [27]. The homeostatic model of insulin resistance (HOMA-IR) index was calculated as glucose × insulin/22.5. Levels of high-sensitivity CRP levels were measured by the clinical biochemistry laboratory. CRP values under the detection limit (<0.17 mg/L) were arbitrarily set at detection limit.

### 2.2. DNA Methylation Analysis

Genomic DNA was isolated from the blood buffy coat using the GenElute Blood Genomic DNA Kit (Sigma, St. Louis, MO, USA) and quantified using both NanoDrop Spectrophotometer (Thermo Scientific, Wilmington, DE, USA) and PicoGreen DNA methods. DNA (1 *μ*g) was bisulfite converted, and quantitative genome-wide methylation analysis was conducted using the Infinium HumanMethylation450 BeadChip (Illumina, San Diego, CA, USA). Arrays were processed at the McGill University and Génome Québec Innovation Centre (Montréal, Canada) according to the manufacturer's instructions (Illumina, San Diego, CA, USA). The Infinium HumanMethylation450 BeadChip array was designed for genome-wide methylation analysis with coverage targeted across gene regions with sites in the promoter region, 5′UTR, first exon, gene body, and 3′UTR. The BeadChip interrogates more than 485 000 methylation sites at single-nucleotide resolution.

Visualization and analysis of methylation data were conducted using the GenomeStudio software version 2011.1 (Illumina Inc.) and the Methylation Module. Methylation levels (beta values; β) were estimated as the ratio of signal intensity of the methylated alleles to the sum of methylated and unmethylated intensity signals of the alleles (β value = C/(T + C)). Internal control probe pairs were used for data correction (background subtraction and normalization), and CpG sites with a detection *P* value >0.05 were removed from analysis. Differences in methylation levels between BMS and AMS groups (mean β values) were tested using Illumina Custom model. False-discovery-rate-corrected (FDR-corrected) *P* values and DiffScores were computed. FDR cutoff of ≤5% was applied to correct for multiple testing. DiffScore is a differential methylation score calculated from *P* values and differences in β (delta β) between the two groups and was used here as the main statistical results for comparison of BMS and AMS siblings. Briefly, a call rate of 99.95% was obtained for methylation data. Differential methylation analysis between BMS and AMS siblings revealed 5698 genes (14 466 probes) with significant differences in methylation levels (FDR-corrected DiffScore ≥|13 | ~*P* ≤ 0.05). Distribution of differentially methylated probes obtained from comparison between BMS and AMS offspring is provided in Supplementary Table 1 available online at http://dx.doi.org/10.1155/2013/492170.

### 2.3. Gene Expression Analysis

Total RNA was isolated and purified from whole blood for 46 offsprings (23 BMS and 23 AMS) using PAXgene Blood RNA Kit (Qiagen) following the manufacturer's recommendations. The integrity of the purified RNA was analyzed using both NanoDrop (Thermo Scientific, Wilmington, DE, USA) and 2100 Bioanalyzer (Agilent Technologies, Cedar Creek, TX, USA). Expression levels were measured using the HumanHT-12 v4 Expression BeadChip (Illumina Inc.) which contains more than 47 000 probes derived from NCBI RefSeq Release 38. Microarray experiments were carried out using 250 ng of total RNA and processed according to the manufacturer's instructions at the McGill University and Génome Québec Innovation Centre (Montréal, Canada). Expression data were visualized and analyzed using the FlexArray software (version 1.6) [28], and the Lumi algorithm was used for expression data analysis and normalization. Probe detection analysis was conducted using the FlexArray filter algorithm to generate a list of expressed probes. To be considered as significantly expressed, a probe had to show a detection *P* value ≤0.05 in at least 25% of the samples of a group. The “Significance Analysis of Microarrays” (SAM) algorithm [29] with unequal-variance (Welch's) *t*-statistic was used to assess differences between BMS and AMS siblings for significantly expressed probes. The SAM algorithm uses permutations of the data set to generate an empirical null distribution and to assess the significance of observed effects, rather than using the hypothetical *t*-distribution. A cutoff of *P* ≤ 0.05 was used to detect differentially expressed probes. In addition, a cutoff of fold change ≥1.2 or ≤0.83 (symmetrical fold change ≥1.2 or ≤−1.2) was used. *P* values obtained from permutations and fold change cutoff values were then used to minimize the chances of false positives. Differential expression analysis revealed a total of 862 probes differentially expressed with a symmetrical fold change ≥|1.2| (*P* ≤ 0.05; fold change <0.83 or >1.2).

Gene expression microarray results were validated using real-time polymerase chain reaction (RT-PCR; Applied Biosystems Gene Expression Assays; Applied Biosystems, Foster City, CA, USA). Spearman correlations were computed for expression levels assessed by microarray and RT-PCR methods in a subset of genes (BCL2, NM_000633; CCND2, NM_001759; NCF2, NM_000433; PIK3R1, NM_181523; PRKCH, NM_006255) for whom correlations between gene expression and methylation levels were observed. Samples were analyzed in duplicate using predesigned probes (Hs00608023_m1, Hs00153380_m1, Hs01084940_m1, Hs00933163_m1 and Hs00178933_m1) and calibrated to the ACTB housekeeping gene (endogenous control; ACTB: Hs99999903_m1). Relative quantification estimations were performed on an Applied Biosystems 7500 Real-Time PCR System (Applied Biosystems, Foster City, CA, USA).

### 2.4. Pathway Analysis

Analysis of potentially altered pathways was conducted using the knowledge base of the Ingenuity Pathway Analysis (IPA) system. The lists of 14 466 differentially methylated and 862 differentially expressed probes produced from differential methylation and differential expression analysis, respectively, were submitted to IPA. Genes from each of these lists were classified according to pathways. IPA measured the likelihood that these genes participate in a particular pathway and calculated *P* values using a right-tailed Fisher's exact test for each pathway. Overrepresented pathways were then obtained from differential methylation and expression analysis.

### 2.5. Statistical Analysis

Clinical data were expressed as mean ± SD. The effect of bariatric surgery in mothers was assessed using a within-subject paired *t*-test. Differences in anthropometric data between BMS and AMS siblings were tested using analysis of variance (general linear model, type III sum of squares) with adjustments for the effects of sex and puberty. In the absence of Tanner scores, we arbitrarily defined puberty as 12 years for girls and 14 for boys. Differences in severe obesity between BMS and AMS siblings were evaluated using BMI percentile and BMI *Z*-score and tested using Fisher's exact test. *P* values for CRP were adjusted for the effects of sex, puberty and BMI percentile, an age- and sex-corrected adiposity measurement. Unadjusted *P* values are also presented. Transformations were applied to nonnormally distributed variables (negative inverse transformed: CRP). Pairwise Pearson correlations between methylation, expression, and plasma CRP were computed for the IL-8 signaling pathway that was overrepresented in both differential methylation and expression analyses. Partial Pearson correlations were also computed for the IL-8 signaling pathway after adjustments for age and sex. Statistical significance was defined as *P* ≤ 0.05. Statistical analyses were conducted using the SAS software version 9.2 (SAS Institute Inc.).

## 3. Results

### 3.1. Impact of Bariatric Surgery in Mothers

A total of 20 mothers (41.0 ± 5.3 years; mean ± SD) who had undergone biliopancreatic diversion were recruited. Mean postoperative follow up was 12 years and 2 months at the time of the study. Bariatric surgery induced dramatic weight loss in mothers. On average, the women weighted 121.5 ± 19.2 kg (BMI = 45.0 ± 7.2) at the time of the surgery and 74.8 ± 11.9 kg (BMI = 27.6 ± 4.8) at the recruitment interview. The mean weight loss of 46.7 ± 16.1 kg was associated with significant improvements in plasma lipids (*P* ≤ 0.01 for TG, HDL-C, LDL-C, total-C, and total-C/HDL-C ratio) and reductions in insulin resistance (HOMA-IR index; *P* ≤ 0.001) and blood pressure (SBP and DBP; *P* ≤ 0.001) with a trend toward lower plasma glucose levels (*P* = 0.06).

### 3.2. Inflammatory Marker in BMS versus AMS Offspring

The 50 BMS and AMS offspring of 20 mothers were aged between 2 years and 8 months and 24 years and 11 months with similar sex distribution (40% males in both BMS and AMS). BMS offspring were born 3 years and 5 months before and AMS offspring were born 3 years and 7 months after maternal bariatric surgery. BMS siblings were older (mean age 14.9 y versus 9.6 y) than AMS siblings (Table 1). Following adjustments for the effects of sex and puberty, AMS offspring showed a trend toward lower body fat percent (*P* = 0.07). There was a significantly lower prevalence of severe obesity in AMS using BMI percentile (*P* = 0.01) or BMI *Z*-score (*P* = 0.05) as indicators. AMS offspring had improved fasting insulin levels (*P* = 0.03), lower HOMA-IR index (*P* = 0.03), and blood pressure. Plasma CRP levels were different in BMS versus AMS offspring (5.14 ± 7.90 versus 3.58 ± 11.09; *P* = 0.03) even after taking into account the sex effect (*P* = 0.03). These differences in plasma CRP levels were no longer significant after further adjustments for the effects of puberty, and BMI (BMI percentile).

### 3.3. Comparison of Pathways Identified by Differential Methylation and Expression Analyses

From the list of 5698 differentially methylated genes between BMS and AMS offspring, IPA revealed 160 pathways significantly overrepresented (*P* < 0.05). Similar analysis conducted from the list of 862 differentially expressed genes between BMS and AMS offspring identified 68 overrepresented pathways. The list of the top 20 overrepresented inflammatory and immune pathways identified from differential methylation analysis along with corresponding results obtained from gene expression analysis is presented in Table 2. Among them, 5 pathways were found to be overrepresented in both methylation and expression: IL-8 signaling, iCOS-iCOSL signaling in T-helper cells, role of NFAT in regulation of the immune response, B-cell receptor signaling, and glucocorticoid receptor signaling.

### 3.4. Correlation of Methylation and Expression Data for the Il-8 Signaling Pathway

Among the 5 pathways listed above, the IL-8 pathway was the most significantly overrepresented from differential methylation analysis. It was thus selected for further analyses to highlight the potential link between gene methylation, expression, and inflammation. Among the 193 genes assigned to the IL-8 signaling pathway by IPA, 70 genes represented by 214 methylation probes were found to be differentially methylated in BMS versus AMS (Supplementary Table 2). Corresponding expression data were available for 102 of these probes (46 genes). Among the 46 genes analyzed, correlations between DNA methylation and expression were significant for 17 genes (23 methylation probes; Table 3). Further analyses were then focused on these 17 genes to correlate gene methylation and expression with CRP levels. There were significant correlations between gene methylation and plasma CRP levels for 16 genes. Gene expression levels of 5 genes were found to correlate with plasma CRP levels (Table 3). All 5 genes with significant correlations between gene expression and plasma CRP levels (*BCL2*, *CCND2*, *NCF2*, *PIK3R1,* and *PRKCH*) also demonstrated correlations between methylation and expression and between gene methylation and plasma CRP levels (Table 3). Following adjustments for the effects of age and sex, similar results were obtained (Supplementary Table 3). All 17 genes initially showing correlation between gene methylation and expression levels demonstrated significant correlation after adjustments. In addition, the *RHOH* gene (methylation probe cg26163153) reached significance level while initially showing a trend toward significant correlation (*P* = 0.09). From the 16 genes correlated with CRP levels, 13 remained significant following adjustments. Only one (*PIK3R1*) of the five genes with significant correlations between gene methylation, expression, and CRP levels remained significant after adjustments, while trends toward significant correlation were found for *BCL2*, *CCND2,* and *NCF2*. Validation of gene expression microarray data for these 5 genes demonstrated significant correlations between microarray and RT-PCR results for 4 of the genes assessed (*CCND2*, *r* = 0.358, *P* = 0.02; *NCF2*, *r* = 0.574, *P* < 0.0001; *PIK3R1*, *r* = 0.349, *P* = 0.02; *PRKCH*, *r* = 0.462, *P* = 0.001), while *BCL2* did not reach significance (*r* = 0.223, *P* = 0.14).

## 4. Discussion

We demonstrated here for the first time that surgical treatment of severe maternal obesity results in sustained differences in the methylome and transcriptome of genes involved in inflammatory pathways in AMS offspring compared to BMS siblings. The proportion of 34.7% of differentially methylated genes (1976 of 5698 genes) being identified by two probes or more strengthen, the validity of our results arguing for a regulation of methylation levels in offspring. Differential analysis of gene methylation and expression levels revealed important differences between the sibling groups. Pathway analysis from differentially methylated genes in BMS versus AMS revealed important regulation of inflammatory and immunity pathways: 29 inflammatory- or immunity-related pathways being identified among the top 50 overrepresented pathways (data not shown). Pathway analysis from differentially expressed genes between BMS and AMS revealed 5 overrepresented pathways among the top overrepresented pathways from differential methylation data (Table 2). Overrepresentation of genes from the IL-8 signaling pathway was identified from both methylation and expression data, and significant correlations were found between gene methylation and expression levels. Moreover, 5 genes from the IL-8 pathway also demonstrated correlation of gene methylation and expression levels with plasma CRP levels. Despite the fact that the impact of DNA methylation on gene expression seems to be site and location dependent, results obtained here for the IL-8 pathway generally comply to the known phenomenon that intragenic methylation (gene body, 5′UTR and 3′UTR) correlates with increased gene expression, while promoter methylation is associated with decreased expression [30, 31].

Gene expression profiling in obese patients who had undergone weight loss or bariatric surgery demonstrated a regulation of inflammation-related transcripts and changes in inflammatory marker levels [32, 33]. In the present study, we extend these data by reporting that metabolic improvements in mothers following biliopancreatic diversion surgery are reflected in gene methylation and expression levels of immunologic and inflammatory genes in offspring, indeed reinforcing the involvement of epigenetic differences in immunity and inflammatory genes in the determination of the offspring phenotypes. A limited number of epigenetic studies have evaluated the impact of maternal nutrition and obesity on methylation of immune and inflammatory genes in offspring. Recently, human studies have revealed the implication of immune and inflammatory gene methylation in children with respect to adiposity. Methylation levels of the *RXRA* gene in umbilical cord tissue DNA of healthy neonates were associated with maternal carbohydrate intake in early pregnancy and childhood adiposity at 9 years old [15]. Relton and collaborators reported associations between anthropometric indices (BMI, fat mass, lean mass, and height) in 9 years old children and methylation levels of selected immune and inflammatory genes in cord blood samples [34]. With a different design involving siblings born under different maternal obesity condition, our study supports the role of epigenetic factors in immunity and inflammatory genes in the determination of offspring phenotypes.

Transcriptomic data revealed that improvements seen in mothers following bariatric surgery are reflected in gene expression levels of immunologic and inflammatory genes in offspring. Animal studies have demonstrated a similar impact of maternal obesity and nutrition on immune and inflammatory genes in the offspring [35–37]. These results are consistent with those obtained from the present gene expression analysis in BMS offspring versus AMS siblings and may provide potential mechanisms for higher levels of systemic inflammation found in BMS offspring.

The present study has several limitations. We used DNA extracted from blood as it is more convenient and acceptable than biopsies of target tissues, especially in children. Although epigenetic signatures have been shown to differ between tissues [38], the overall impact is minor given the known similarities in methylation patterns [39]. Gene expression levels are also known to be tissue-or cell-type dependent but also correlated [40–42] thus justifying the use of blood in clinical studies. Differential gene expression analysis led to a limited number of differentially expressed genes (862) in comparison with the 5698 differentially methylated genes identified. This discrepancy may be attributable to differences in statistical algorithms, in corrections for multiple testing, or in the different cutoffs used; a cutoff fold change ≥1.2 or ≤0.83 was applied for gene expression analysis. Nonetheless, pathway analyses conducted from differentially methylated and expressed genes are concordant and demonstrate an overrepresentation of immune and inflammatory pathways. We did not stratify for sex in these gene methylation and expression analyses; several studies did not find sex-specific autosomal methylation patterns [43, 44]. Owing to the before-after design of the study, we were unable to fully correct for the age difference between the sibling groups. This design and the young age of the AMS offspring (80% being younger than 12 years old) do not allow discussing the long-term effects in children. Longitudinal studies are thus needed to further assess long-term effects of maternal bariatric surgery in the offspring. Age-related differential methylation has been reported but was shown to be site- and location dependent [45–47] and represented a very small proportion of CpG sites [47]. Furthermore, longitudinal studies in humans demonstrated relative stability of DNA methylation over time [39]. In regards to the general conclusion drawn from our results, various factors may be perceived as potential bias toward inflammatory effects. The potential impact of maternal weight regain on offspring is limited in the current study considering the sustained weight loss achieved with the biliopancreatic diversion with duodenal switch [19, 48, 49]. Analysis of maternal clinical files along with the lack of inflammation-related perioperative complications in biliopancreatic diversion with duodenal switches [48] argues against the potential impact of specific inflammatory condition in mothers. The current study was not designed to analyze methylation and expression changes induced in mothers following bariatric surgery. However, based on a recent meta-analysis demonstrating a reduction in low-grade inflammation associated with obesity following bariatric surgery [22], it would be tempting to speculate that these changes may be accompanied by similar changes in methylation and expression levels. Improvements in inflammatory profiles in mothers following bariatric surgery may be potentially responsible for the inflammatory effects seen in offspring through modification of the *in utero* environment and improved methylation profiles in AMS offspring.

## 5. Conclusion

Using analysis of gene methylation and expression data, we reveal that improvements seen in mothers following bariatric surgery reflect on the expression and methylation levels of genes involved in immune and inflammatory pathways in the offspring. These results argue for a beneficial effect of maternal weight loss before pregnancy and provide potential mechanisms of action for physiological improvements through regulation of methylation and expression of genes involved in inflammatory and immune pathways. Since inflammation is recognized as an independent risk factor for cardiovascular diseases (CVD) and is also associated with the development of the metabolic syndrome, insulin resistance, hypertension, and obesity [50, 51], it would be important to conduct longitudinal studies to examine the long-term effect of maternal bariatric surgery on offspring.

## Supplementary Material

Supplementary Table 1: Distribution of differentially methylated probes obtained from comparison between BMS and AMS offspring.
Supplementary Table 2: List of differentially methylated genes from the IL-8 signaling pathway.
Supplementary Table 3: Partial correlations of gene methylation and expression with hsCRP levels following adjustment for the effects of age and sex.

## Figures and Tables

**Table 1:** Offspring characteristics.

	BMS	AMS	*P*-values^1^
*N* (males)	25 (10)	25 (10)	
Age (years)	14.9 ± 6.2	9.6 ± 5.3	0.002
Anthropometric data			
BMI percentile	69.2 ± 40.7	65.7 ± 32.6	0.80
BMI *Z*-score	1.84 ± 2.08	0.85 ± 1.47	0.22
Fat percent^2^	29.9 ± 13.9	21.4 ± 10.3	0.07
Severe obesity			
BMI percentile > 98% (*N*)	12	3	0.01
BMI *Z*-score > 3 (*N*)	7	1	0.05
Blood pressure			
SBP (mm Hg)	111.3 ± 14.7	97.4 ± 14.6	0.01
DBP (mm Hg)	64.6 ± 9.8	53.4 ± 12.8	0.002
Lipid profile^3^			
Total-C (mmol/L)	4.43 ± 0.66	4.21 ± 0.58	0.24
LDL-C (mmol/L)	2.65 ± 0.55	2.53 ± 0.58	0.53
HDL-C (mmol/L)	1.30 ± 0.29	1.30 ± 0.25	0.54
TG (mmol/L)	1.04 ± 0.43	0.82 ± 0.37	0.21
Total-C/HDL-C	3.56 ± 0.93	3.37 ± 0.85	0.87
Glucose metabolism^3^			
Fasting glucose (mmol/L)	4.92 ± 0.43	4.71 ± 0.43	0.22
Insulin (*μ*U/mL)	18.82 ± 12.20	11.27 ± 7.36*	0.03
Homa-IR	4.27 ± 3.23	2.43 ± 1.72*	0.03

Values are presented as mean ± SD.

^1^ *P*-values adjusted for sex and puberty except for BMI percentile and BMI *Z*-score.

^2^Fat percent at 6 years or more (BMS, *N* = 23; AMS, *N* = 17).

^3^BMS, *N* = 25; AMS, *N* = 21.

Abbreviations: AMS: after maternal surgery; BMS: before maternal surgery; BMI: body mass index; SBP and DBP: systolic diastolic and systolic blood pressure; Total-C: total cholesterol; LDL-C: low-density lipoprotein cholesterol; HDL-C: high-density lipoprotein cholesterol; TG: triglycerides.

**Table 2:** Comparison of the top 25 overrepresented canonical immune/inflammatory pathways identified from differential methylation and expression analyses between BMS and AMS siblings.

IPA canonical pathways	*P* value	*N* of diff. meth. genes	*P*-value	*N* of Diff. Express. genes
CD28 signaling in T-helper cells	4.24 × 10^−7^	54	0.40	5
Leukocyte extravasation signaling	6.34 × 10^−7^	78	0.25	13
Role of NFAT in cardiac hypertrophy	6.98 × 10^−7^	76	NI	—
T-Cell receptor signaling	7.55 × 10^−7^	47	0.07	7
Dendritic cell maturation	8.22 × 10^−7^	66	0.10	9
Clathrin-mediated endocytosis signaling	1.24 × 10^−6^	74	0.09	11
**IL-8 signaling**	2.44 × 10^−6^	**70**	**0.03**	**12**
**iCOS-iCOSL signaling in T-helper cells**	5.35 × 10^−6^	**48**	**0.01**	**9**
Virus entry via endocytic pathways	5.48 × 10^−6^	43	NI	—
NF-*κ*B signaling	6.08 × 10^−6^	66	0.08	10
**Role of NFAT in regulation of the immune response**	8.14 × 10^−6^	**69**	**0.02**	**12**
PKC*θ* signaling in T lymphocytes	9.18 × 10^−6^	50	0.24	6
Macropinocytosis signaling	2.5 × 10^−5^	32	NI	—
CCR5 signaling in macrophages	2.66 × 10^−5^	34	0.42	3
CTLA4 Signaling in Cytotoxic T Lymphocytes	2.71 × 10^−5^	40	NI	—
**B-Cell receptor signaling**	2.96 × 10^−5^	**58**	**0.008**	**12**
fMLP signaling in neutrophils	3.94 × 10^−5^	47	0.05	8
RAR activation	4.32 × 10^−5^	66	0.32	8
IL-1 signaling	6.55 × 10^−5^	40	0.27	5
**Glucocorticoid receptor signaling**	6.72 × 10^−5^	**92**	**0.005**	**19**

Pathways in bold are overrepresented in both gene methylation and expression data. Abbreviations: diff. express.: differentially expressed; diff. Meth.: differentially methylated; *N*: number; NI: not identified.

**Table 3:** Correlations of gene methylation and expression with CRP levels for genes from the IL-8 signaling pathway.

Gene symbol	Methylation probe^1^	Localization	Transcript accession number	Gene methylation versus expression (N = 46)		Gene methylation versus hsCRP level (*N* = 50)		Gene expression versus hsCRP level (*N* = 45)	
				Pearson	*P* value	Pearson	*P* value	Pearson	*P* value
BCL2	**cg08223235**	Gene body	NM_000633	0.451	0.002	−0.541	<0.0001	−0.337	0.02
CCND2	**cg27317813**	Gene body	NM_001759	0.556	<0.0001	−0.527	<0.0001	−0.360	0.02
CXCR1	cg13048967	5′UTR	NM_000634	−0.431	0.003	−0.452	0.001	0.209	0.17
CXCR1	cg15768138	5′UTR	NM_000634	−0.440	0.002	−0.482	0.0004	0.209	0.17
GNA12	cg01139696	Gene body	NM_007353	0.345	0.02	0.143	0.32	0.148	0.33
GNA12	cg06544239	Gene body	NM_007353	0.415	0.004	0.145	0.32	0.148	0.33
GNA13	cg25554496	Gene body	NM_006572	−0.371	0.01	0.497	0.0002	−0.260	0.08
GNAI3	cg19244300	Gene body	NM_006496	0.300	0.04	−0.500	0.0002	−0.143	0.35
GNB2L1	cg01919999	Gene body	NM_006098	0.430	0.003	−0.420	0.002	−0.177	0.25
GNG2	cg27019717	5′UTR	NM_053064	0.414	0.004	−0.523	<0.0001	−0.213	0.12
GNG7	cg04603130	5′UTR	NM_052847	0.395	0.007	−0.545	<0.0001	−0.250	0.10
GNG7	cg24339704	5′UTR	NM_052847	0.393	0.007	−0.524	<0.0001	−0.250	0.10
GNG7	cg26988138	5′UTR	NM_052847	0.296	0.05	−0.369	0.008	−0.250	0.10
ITGAX	cg04742550	TSS200	NM_000887	−0.315	0.03	−0.352	0.01	0.133	0.38
LIMK2	cg07713946	3′UTR	NM_016733	0.325	0.03	0.440	0.001	0.278	0.06
MAP4K4	cg13522882	Gene body	NM_145687	−0.309	0.04	0.483	0.0004	−0.162	0.29
NCF2	**cg09076123**	5′UTR	NM_000433	−0.304	0.04	−0.508	0.0002	0.323	0.03
NCF2	**cg09472600**	Gene body	NM_000433	−0.395	0.007	−0.529	<0.0001	0.323	0.03
PIK3C2B	cg21195376	5′UTR	NM_002646	−0.293	0.05	0.479	0.0004	−0.166	0.28
PIK3R1	**cg00067720**	TSS1500	NM_181523	0.475	0.0008	−0.566	<0.0001	−0.372	0.01
PRKCA	cg14648237	Gene body	NM_002737	0.424	0.003	−0.518	0.0001	−0.279	0.06
PRKCA	cg22435313	Gene body	NM_002737	−0.374	0.01	0.562	<0.0001	−0.279	0.06
PRKCH	**cg14001486**	Gene body	NM_006255	−0.564	<0.0001	0.573	<0.0001	−0.311	0.04

^1^Bold: significantly correlate in all 3 analyses.
