# Supplementary material for: Methylation and Expression of Immune and Inflammatory Genes in the Offspring of Bariatric Bypass Surgery Patients
Source: J Obes. 2013 Jun 11;2013:492170. doi: 10.1155/2013/492170 (PMC3693160; doi:10.1155/2013/492170)
Supplement: Supplementary file 1 — Supplementary Table 1: Distribution of differentially methylated probes obtained from comparison between BMS and AMS offspring. Supplementary Table 2: List of differentially methylated genes from the IL-8 signaling pathway. Supplementary Table 3: Partial correlations of gene methylation and expression with hsCRP levels following adjustment for the effects of age and sex. [file 492170.f1.pdf]

Supplementary Table 1. Distribution of differentially methylated probes obtained from comparison between BMS and AMS offspring.

	**N**
Number of differentially methylated probes	14 466
Localization*	
Gene body	5746
3'UTR	714
1st exon	273
5'UTR	1145
Promoter region**	2292
TSS1500	1561
TSS200	731
Intergenic region	4296

* Localization relative to the 1st transcript annotated for each CpG site and provided for the Infinium HumanMethylation450 BeadChip.
** Promoter region including TSS1500 (within 1500 bps of a TSS) and TSS200 (within 200 bps of a TSS).
Abbreviations: AMS, after maternal surgery; BMS, before maternal surgery; TSS, transcription start site; N, number

Supplementary Table 2. List of differentially methylated genes from the IL-8 signaling pathway.

Pathway	Genes
IL-8 signaling	AKT1, ANGPT1, ANGPT2, AZU1, BCL2, CCND2, CCND3, CDH1, CXCR1, CXCR2, DEFA1, FLT1, GNA12, GNA13, GNAI1, GNAI2, GNAI3, GNAS, GNB1, GNB2L1, GNB5, GNG10, GNG12, GNG2, GNG4, GNG7, GPLD1, IKBKB, IKBKG, IL9, IRAK2, IRAK3, ITGAX, ITGB2, KDR, KRAS, LIMK2, MAP4K4, MAPK10, MAPK9, MPO, NCF2, NOX1, PAK2, PDGFC, PIK3C2B, PIK3C3, PIK3CA, PIK3CD, PIK3CG, PIK3R1, PIK3R5, PIK3R6, PLCB2, PLD1, PLD3, PRKCA, PRKCD, PRKCE, PRKCH, PRKCQ, PRKCZ, PRKD1, PRKD3, PTK2, RHOG, RHOH, ROCK1, SRC, VCAM1

Supplementary Table 3. Significant partial correlations of gene methylation and expression with hsCRP levels following adjustment for the effects of age and sex.

Gene symbol	Methylation probe[1]	Localization	Transcript accession number	Gene methylation vs expression (N=46)		Gene methylation vs hsCRP level (N=50)		Gene expression vs hsCRP level (N=45)	
				Pearson	p-value	Pearson	p-value	Pearson	p-value
BCL2	cg08223235	Gene body	NM_000633	0.428	0.004	-0.408	0.004	-0.276	0.07
CCND2	cg27317813	Gene body	NM_001759	0.536	0.0002	-0.406	0.004	-0.264	0.09
CXCR1	cg13048967	5'UTR	NM_000634	-0.421	0.004	-0.291	0.05	0.172	0.27
CXCR1	cg15768138	5'UTR	NM_000634	-0.428	0.004	-0.315	0.03	0.172	0.27
GNA12	cg01139696	Gene body	NM_007353	0.338	0.02	0.219	0.13	0.203	0.19
GNA12	cg06544239	Gene body	NM_007353	0.414	0.005	0.141	0.34	0.203	0.19
GNA12	cg08377331	Gene body	NM_007353	0.339	0.02	0.076	0.61	0.203	0.19
GNA12	cg12444411	Gene body	NM_007353	0.304	0.04	0.060	0.69	0.203	0.19
GNA12	cg19524238	Gene body	NM_007353	0.340	0.02	0.123	0.41	0.203	0.19
GNA13	cg25554496	Gene body	NM_006572	-0.382	0.01	0.350	0.01	-0.149	0.34
GNAI3	cg19244300	Gene body	NM_006496	0.304	0.04	-0.345	0.02	-0.031	0.84
GNAS	cg24617313	TSS1500	NM_080425	0.311	0.04	0.082	0.58	-0.106	0.50
GNB2L1	cg01919999	Gene body	NM_006098	0.461	0.002	-0.225	0.12	-0.060	0.70
GNG2	cg27019717	5'UTR	NM_053064	0.412	0.006	-0.388	0.006	-0.098	0.53
GNG7	cg04603130	5'UTR	NM_052847	0.369	0.01	-0.418	0.003	-0.169	0.28
GNG7	cg24339704	5'UTR	NM_052847	0.364	0.02	-0.380	0.008	-0.169	0.28
ITGAX	cg04742550	TSS200	NM_000887	-0.335	0.03	-0.130	0.38	0.101	0.52
LIMK2	cg07713946	3'UTR	NM_016733	0.329	0.03	0.269	0.06	0.253	0.10
MAP4K4	cg13522882	Gene body	NM_145687	-0.298	0.05	0.335	0.02	-0.073	0.64
NCF2	cg09076123	5'UTR	NM_000433	-0.279	0.07	-0.355	0.01	0.276	0.07
NCF2	cg09472600	Gene body	NM_000433	-0.368	0.01	-0.400	0.005	0.276	0.07
PIK3C2B	cg21195376	5'UTR	NM_002646	-0.284	0.06	0.310	0.03	-0.039	0.81
PIK3R1	<u>cg00067720</u>	TSS1500	NM_181523	0.466	0.001	-0.446	0.002	-0.309	0.04
PRKCA	cg14648237	Gene body	NM_002737	0.433	0.003	-0.368	0.01	-0.233	0.13
PRKCA	cg22435313	Gene body	NM_002737	-0.368	0.01	0.451	0.001	-0.233	0.13
PRKCH	cg14001486	Gene body	NM_006255	-0.557	<0.0001	0.455	0.001	-0.209	0.18
RHOH	cg26163153	TSS1500	NM_004310	-0.303	0.05	0.331	0.02	-0.120	0.44

[1] Underlined: significantly correlate in all 3 analyses.
